# Epidemiological profile of people with accidental tetanus admitted to the intensive care unit of a public hospital: a retrospective study, Fortaleza, 2017-2024

**DOI:** 10.1590/S2237-96222026v35e20250232.en

**Published:** 2026-04-04

**Authors:** Lara Figueiredo Vasconcelos, Francisco Cleiton Ribeiro Freitas, João Pedro Nunes Leiros, Carlos Heitor Ribeiro dos Santos, Caio Douglas Guilherme Rodrigues, Riany De Sousa Sena

**Affiliations:** 1 Hospital São José de Doenças Infecciosas, Fortaleza, CE, Brazil Hospital São José de Doenças Infecciosas Fortaleza CE Brazil; 2 Hospital Geral Dr. Waldemar Alcântara, Fortaleza, CE, Brazil Hospital Geral Dr. Waldemar Alcântara Fortaleza CE Brazil; 3 Universidade de Fortaleza, Fortaleza, CE, Brazil Universidade de Fortaleza Fortaleza CE Brazil

**Keywords:** Tetanus, Intensive Care Units, Health Profile, Hospitals, Specialized, Retrospective Studies., Tétanos, Unidades de Cuidados Intensivos, Perfil de Salud, Hospitales Especializados, Estudios Retrospectivos

## Abstract

**Objective:**

To analyze the epidemiological profile of people with accidental tetanus admitted to an intensive care unit in Fortaleza from 2017 to 2024.

**Methods:**

This was a retrospective cohort study conducted in a public hospital in Ceará, Brazil. Individuals aged ≥18 years with a confirmed diagnosis of tetanus, regardless of sex, admitted to the intensive care unit between January 2017 and May 2024, were included. One medical record with insufficient sociodemographic and clinical data was excluded.

**Results:**

A total of 44 participants were included in the study, most of whom were male (n=41), predominantly aged 20-40 years (n=20); married (n=19); self-identified as Brown (Brazilian mixed race) (n=40); and had incomplete elementary education (n=20). Most resided in Fortaleza (n=18). Workers in the agricultural and livestock sector (n=14) were the most represented occupational group. The most frequent entry site was the feet (n=15). Most participants were unaware of their vaccination status (n=26). Being 60 years or older (odds ratio OR 11.6; 95% confidence interval 95%CI 1.9; 70.2; p-value 0.003) and smoking (OR 9.8; 95%CI 1.1; 88.2; p-value 0.019) were significantly associated with death as an outcome.

**Conclusion:**

The majority of participants were working-age men with incomplete elementary education, primarily employed in the agricultural and livestock sectors. Being 60 years of age or older and smoking were significantly associated with a higher likelihood of death.

## Resumen

**Objetivo:**

Analizar el perfil epidemiológico de personas con tétanos accidental internadas en una unidad de cuidados intensivos en Fortaleza entre 2017 y 2024.

**Métodos:**

Se trató de un estudio de cohorte retrospectivo realizado en un hospital público de Ceará, Brasil. Se incluyeron individuos de 18 años o más, con diagnóstico confirmado de tétanos, independientemente del sexo, internados en la unidad de cuidados intensivos entre enero de 2017 y mayo de 2024. Se excluyó una historia clínica con datos sociodemográficos y clínicos insuficientes.

**Resultados:**

Se incluyeron en el estudio 44 participantes, la mayoría hombres (n=41), predominantemente de 20 a 40 años (n=20); casados (n=19), autoidentificados como de raza/cor de piel parda (n=40), y con enseñanza primaria incom­pleta (n=20). La mayoría residía en Fortaleza (n=18). Los trabajadores del sector agropecuario (n=14) fueron el grupo ocupacional más representado. El sitio de entrada más frecuente fueron los pies (n=15). La mayoría desconocía su estado vacunal (n=26). Tener 60 años o más (razón de momios OR 11,6; intervalo de confianza del 95% IC95% 1,9; 70,2; valor de p 0,003) y el tabaquismo (OR 9,8; IC95% 1,1; 88,2; valor de p 0,019) se asociaron significativamente con el desenlace de muerte.

**Conclusión:**

La mayoría de los participantes eran hombres en edad laboral, con enseñanza primaria incompleta y trabajadores del sector agropecuario. Tener 60 años o más y el tabaquismo se asociaron significativamente con mayor probabilidad de muerte.

**Ethical aspects**

This research respected ethical principles, having obtained the following approval data:

Research ethics committee: Hospital São José de Doenças Infecciosas

Opinion number: 6847296

Approval date: 24/6/2024

Certificate of submission for ethical appraisal: 78330624.2.0000.5044

Informed consent record: Dismissed.

## Introduction 

Accidental tetanus is a severe, non-communicable infection resulting from the action of exotoxins released by the bacterium *Clostridium tetani*^1^. Tetanus continues to be a considerable cause of morbidity and mortality in developing and underdeveloped nations, despite a global decline in incidence, particularly in regions with limited access to vaccination and health services ^2^. Between 2014 and 2024, Brazil recorded 2,385 confirmed cases of accidental tetanus, with the highest incidence in the Northeast (33.2%), Southeast (21.4%), and South (21.3%) regions. In 2022, 2023, and 2024, the case-fatality rate in Brazil was 26.6%, 29.2%, and 26.9%, respectively. These figures are substantially higher than those observed in developed countries such as Japan (6.8% between 2010 and 2016) and the United States (13.2% between 2001 and 2008) ^3^^-^^5^.

Immunity against tetanus is acquired through vaccination. According to the Brazilian National Immunization Program, the Brazilian National Health System (SUS) provides a tetanus vaccination schedule beginning with three doses in the first year of life, a booster at 15 months, and another booster at 4 years of age. From then on, vaccination is administered every ten years, or every five years in case of severe injuries ^6^. Despite being preventable through vaccination, tetanus can still progress to severe forms, requiring admission to an intensive care unit (ICU), especially among more vulnerable populations ^2^^,^^7^.

To treat accidental tetanus, it is recommended that moderate and severe forms be managed in the ICU for continuous monitoring and intensive treatment by a trained multidisciplinary team, particularly when risk factors for poor prognosis, such as dysautonomia, are present ^8^. In Brazil, between 2007 and 2016, the average length of hospital stay due to accidental tetanus was 17.1 days, with an average cost of BRL 5,022.32 per admission and estimated annual expenses of BRL 9,852,868.52 ^9^. Accidental tetanus has a high case-fatality rate, which contributes to an increase in the number of hospitalizations and, consequently, high costs, overburdening the health system ^10^.

The persistence of severe cases reveals significant deficits in Primary Health Care, such as failures in active surveillance and health education. A variety of structural and contextual barriers-such as distance to healthcare facilities, transportation difficulties, and disarticulation between levels of care-compromise the effectiveness of health promotion and prevention initiatives. It becomes imperative to develop health planning that recognizes the territorial and sociocultural specificities of users, enabling the formulation of strategies focused on expanding access and achieving comprehensive care ^11^.

Vulnerability to tetanus is not homogeneously distributed and varies according to occupational, environmental, and socioeconomic factors ^2^. In Brazil, accidental tetanus has shown a tendency to disproportionately affect the elderly population and rural workers, due to lower vaccination coverage and greater vulnerability to accidents. This is evidenced by the mortality rate, which has decreased across all age groups, except among those over 60 years old ^12^. 

As disease incidence declines, shifts in the epidemiological profile may occur, necessitating continuous evaluation to guide public policies and preventive strategies ^2^. Although it is a disease that can be effectively prevented through vaccination and ongoing, accessible, low-cost health education, accidental tetanus remains a public health problem with systemic, economic, and social repercussions ^2^. Thus, a better understanding of the profile of people affected by tetanus may help guide the planning and implementation of public health and epidemiological surveillance actions, as well as preventive strategies and vaccination campaigns. 

The objective of this study was to analyze the epidemiological profile of people with accidental tetanus admitted to the ICU of a public hospital in Fortaleza between 2017 and 2024.

## Methods

Design

This retrospective cohort study is based on medical record review, conducted in a public referral hospital for infectious diseases in Ceará, Brazil.

Setting

The study was conducted between June 2023 and January 2025 at a public hospital in Ceará, a referral center for infectious diseases and part of the Brazilian National Health System (SUS), with a history of involvement in controlling serious infectious and endemic diseases in the state ^13^. In 2025, the institution had 120 beds, distributed across six inpatient units and one ICU. 

Participants

The sample included individuals with confirmed diagnoses of accidental tetanus, registered in the epidemiological surveillance unit's notification forms. The diagnosis was established based on clinical manifestations, such as trismus, nuchal rigidity, muscle spasms, *risus sardonicus*, abdominal rigidity, opisthotonos, and limb rigidity. Other inclusion criteria were: age 18 years or older, sex (male or female), and a need for ICU admission between January 2017 and May 2024. One medical record with insufficient sociodemographic and clinical data for collection was excluded. 

Variables

Sociodemographic and clinical variables collected from medical records included age, sex, marital status, race/skin color, educational level, place of residence, occupation, body mass index, and presence of comorbidities. Data related to tetanus included portal of entry, vaccination status, incubation period, symptom duration, and clinical outcome (death or hospital discharge).

Data sources 

Data were obtained from epidemiological surveillance notification forms and hospital records. Information was collected using a data collection form, completed by a health professional, and organized in an Excel spreadsheet for subsequent analysis. 

Bias 

The study's retrospective design introduces a potential bias in data collection, given that clinical records are not always complete, standardized, or accurate. Potential omissions or recording errors may compromise the quality of the analyzed data. 

Study size

The sample was non-random, based on data available in the electronic medical records during the initial screening of participants.

Statistical methods 

Data were analyzed using the Statistical Package for the Social Sciences (SPSS, Chicago, United States, version 24). Initially, a descriptive analysis was performed, including absolute and relative frequencies for categorical variables, as well as mean, standard deviation, median, and interquartile range for numerical variables. Normality of distribution was verified using the Shapiro-Wilk test. To evaluate the determinants of the outcome (death), measures of association were calculated using odds ratios (OR) with respective 95% confidence intervals (95%CI). A p-value<0.05 was considered statistically significant. 

## Results

The hospital's epidemiological surveillance unit reported sixty-two individuals; of these, 18 cases were excluded (Figure 1). The final sample of the study consisted of 44 individuals.

The sociodemographic characteristics of the study participants are presented in Table 1. Most participants were male (n=41) and belonged to the age groups 40-59 years (n=20) and 60 years or older (n=17). They were predominantly married (n=19), self-identified as Brown (n=40), and had incomplete elementary education (n=20). The sample was predominantly composed of residents from Fortaleza (n=18) and the countryside of Ceará (n=17). Workers in the agricultural and livestock sector (n=14) and unemployed individuals (n=10) were the most represented occupational groups. The mean body mass index of participants was 24.0 (20.7 kg/m²-26.0 kg/m²) kg/m². The majority reported alcohol use disorder (n=27), while half of the participants were smokers (n=22). The mean number of comorbidities was 0.9±1.1, the most prevalent being hypertension (n=13), diabetes (n=6), and chronic obstructive pulmonary disease (n=4). 

**Figure 1:**
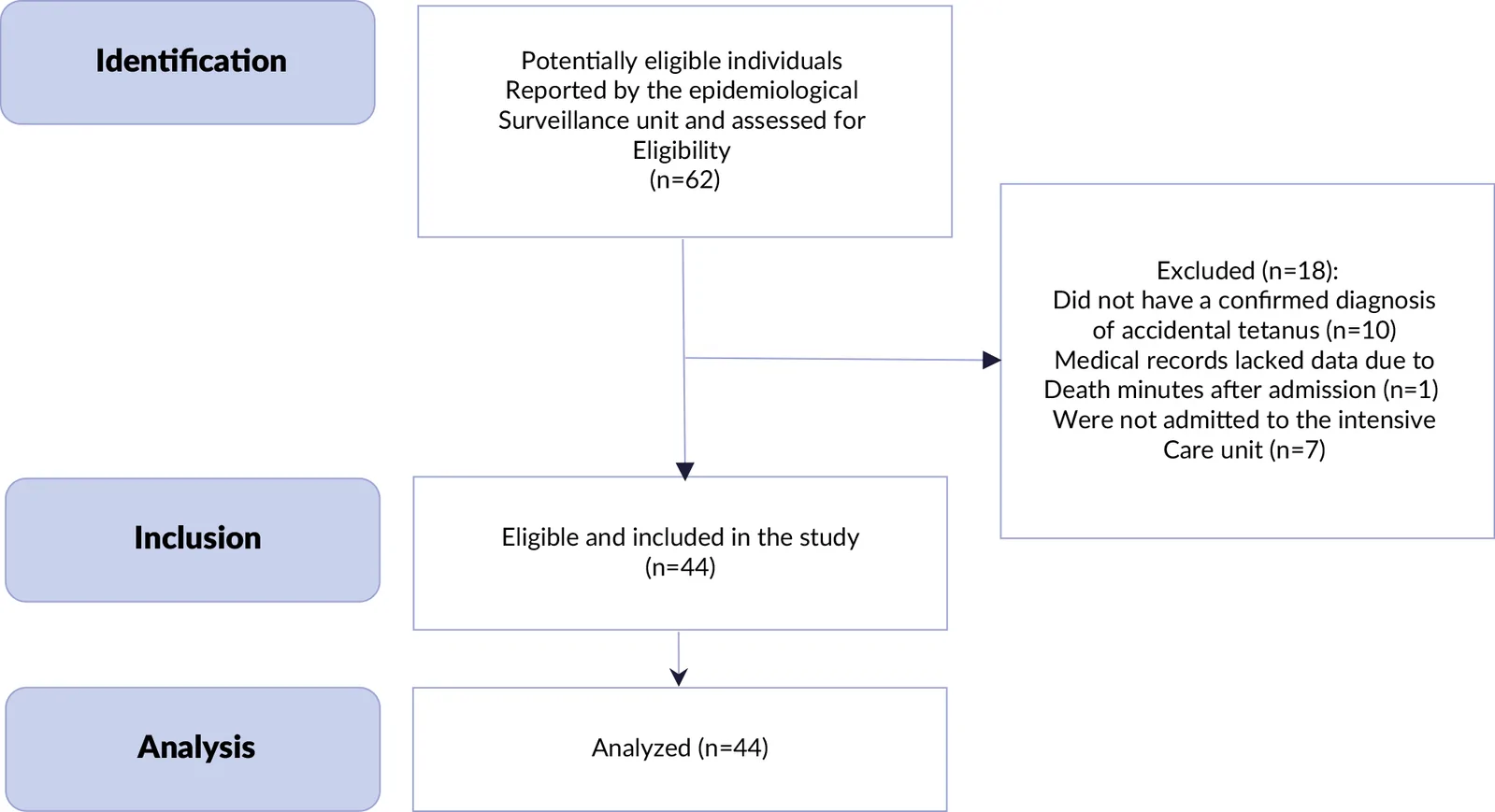
Flowchart of participant inclusion in the study. Fortaleza, 2017-2024 (n=62

**Table 1:** Sociodemographic characteristics of individuals with severe accidental tetanus admitted to the intensive care unit. Fortaleza, 2017-2024 (n=44)

Variables	n
Male	41
Age group (years)	
20-39	7
40-59	20
≥60	17
Marital Status	
Married	19
Single	14
Not informed	6
Divorced	3
Widowed	2
Race/skin color	
Brown	40
Black	3
White	1
Education level	
Incomplete elementary school	20
Illiterate	11
Literate	3
Not informed	3
Complete elementary school	3
Incomplete high school	2
Complete high school	2
Home	
Fortaleza	18
Countryside of Ceará	17
Metropolitan region of Fortaleza	8
Other Brazilian states	1
Occupation	
Agriculture/Livestock	14
Unemployed	10
Construction worker	5
Salesperson	5
Independent contractor	2
Retired	2
Waste collector	2
General services worker	2
Baker	1
Domestic worker	1
Alcohol use disorder	27
Smoking habits	22
Comorbidities	
Hypertension	13
Diabetes	6
Chronic obstructive pulmonary disease	4
Chronic low back pain	2
Pulmonary emphysema	2
Chronic renal failure	2

The clinical characteristics related to accidental tetanus are presented in Table 2. The most frequent portals of entry were the feet (n=15) and legs (n=10), followed by cases where the site was undefined (n=7). The majority (n=26) were unaware of their vaccination status, while 11 reported not having received any vaccinations, and seven had incomplete vaccination schedules. The median incubation period was 10 days (interquartile range 7-15 days), and the median time from symptom onset to hospital admission was three days (interquartile range 2-6 days). Symptoms recorded on admission included muscle spasms (n=41), trismus (n=40), dysphagia (n=39), dysarthria (n=39), limb rigidity (n=33), abdominal rigidity (n=29), nuchal rigidity (n=27), opisthotonos (n=13), and *risus sardonicus* (n=6). Regarding hospital outcomes, 34 individuals were discharged, eight died, and two were transferred to another institution.

The trend in tetanus cases and tetanus-related deaths in Fortaleza from 2017 to 2024 is shown in Figure 2. A peak of tetanus cases occurred in 2022, with nine records, followed by a decrease in the subsequent two years. As for deaths, 2022 also recorded the highest number, with four deaths. Throughout the study period, variations were observed in both the absolute number of cases and case-fatality rates, with a particular emphasis on 2020 and 2024. Recently, although the number of cases was relatively low, the proportion of deaths relative to cases was high.

Being 60 years or older (OR 11.6; 95%CI 1.9; 70.2; p-value 0.003) and smoking (OR 9.8; 95%CI 1.1; 88.2; p-value 0.019) were significantly associated with death as an outcome Table 3.

**Table 2:** Characteristics related to individuals with accidental tetanus admitted to the intensive care unit. Fortaleza, 2017-2024 (n=44)

Variables	n
Portal of entry	
Feet	15
Legs	10
Unknown	7
Dental	4
Hands	3
Arms	3
Head/neck	1
Backbone	1
Vaccination schedule	
Not informed/unknown	26
Unvaccinated	11
Incomplete schedule	7
Symptoms on Admission	
Muscle spasms	41
Trismus	40
Dysphagia	39
Dysarthria	39
Limb rigidity	33
Abdominal rigidity	29
Nuchal rigidity	27
Opisthotonos	13
Risus sardonicus	6
Clinical outcome during hospitalization	
Hospital discharge	34
Death	8
Transfer to another institution	2

**Figure 2:**
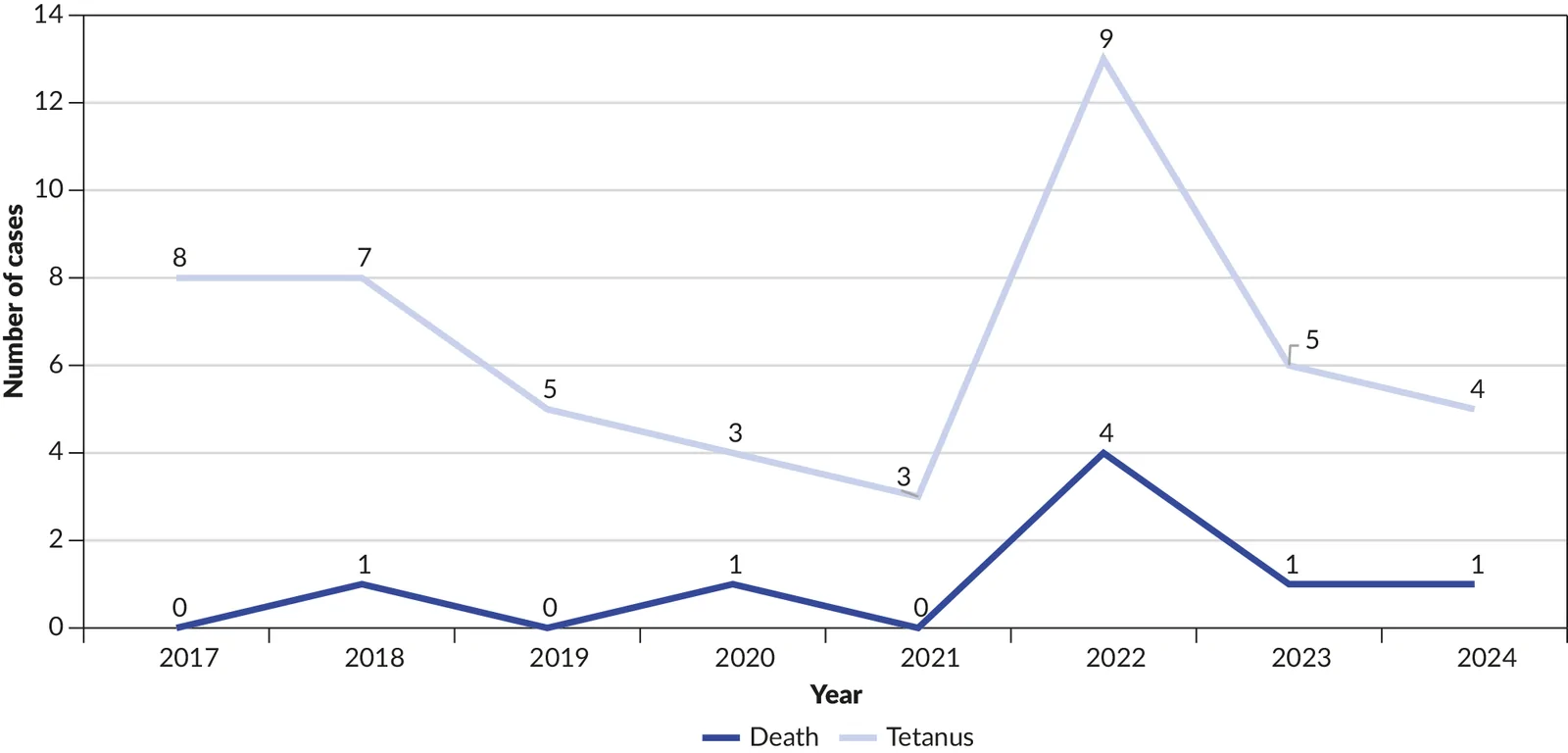
Number of tetanus cases and tetanus-related deaths. Fortaleza, 2017-2024

**Table 3:** Odds ratios (OR) and 95% confidence intervals (95%CI) for death from accidental tetanus, according to study variables. Fortaleza, 2017-2024 (n=42)

Variables	Death	Hospital discharge	OR	(95%CI)	p-value
Age (years)					
≥60	6	7	11.6	(1.9; 70.2)	0.003
<60	2	27			
Sex			2.3	(0.2; 28.9)	0.513
Female	1	2			
Male	7	32			
Smoking habits					
Yes	4	5	9.8	(1.1; 88.2)	0.019
No	4	30			
Duration of symptoms (days)					
≥7	4	16	3.4	(0.3; 42.4)	0.337
<7	2	10			
Use of neuromuscular blocking agents					
Yes	8	23	1.3	(1.09; 1.6)	0.063
No	0	11			
Dysautonomia			1.8	(0.2; 17.3)	0.600
Yes	3	5			
No	5	29			

## Discussion

In this study, severe accidental tetanus predominantly affected men and individuals aged 40-59 years or 60 years and older. Most of these individuals were residents of Fortaleza. They were predominantly married, self-identified as Brown, had incomplete elementary education, and were employed in the agriculture and livestock sector. Most were unaware of their vaccination status or were not vaccinated against tetanus, and also reported alcohol use disorder and smoking habits. The most frequent portals of entry for tetanus were the feet and legs; in several cases, the site of entry could not be defined. Symptoms included muscle spasms, trismus, dysphagia, and dysarthria. Being 60 years of age or older and smoking were significantly associated with a higher likelihood of death.

The profile of the individuals in this study was similar to that described in other cities in the Northeast, such as in Alagoas between 2007 and 2017 ^14^, where most cases occurred in males (91.0%), in the 18-49 age group (67.2%), agriculture en livestock workers (27.8%), and those residing in urban areas (83.1%). Between 2006 and 2018 in Bahia ^15^, most cases also occurred in males (83.3%), aged 35-49 years (30.3%), who self-identified as Brown (38.6%), had incomplete elementary education (30.3%), and were residents of the state capital (33.3%). The most predominant occupations in this locality were farmers (n=36, 27.3%) and construction workers (n=26, 19.7%). However, in this study, the majority of records in the sample lacked occupation data (37.9%), introducing bias into the results. Despite Brazil's socioeconomic heterogeneity, the profile of individuals affected by accidental tetanus between 1990 and 2009 in São Paulo ^12^ and between 2008 and 2018 in Santa Catarina ^16^ was similar to that observed in the present study. 

The profile here differs from those observed in other countries, such as South Korea, France, and Italy, where the highest incidence of accidental tetanus occurs among females. The likely explanation for this disparity is the higher vaccination coverage in males, achieved through compulsory military service ^17^.

The higher prevalence of the disease among males in Brazil can be attributed to several factors, the foremost being low adherence to vaccination campaigns. This scenario results in suboptimal vaccination coverage, which in turn contributes to greater vulnerability to preventable diseases such as tetanus. Furthermore, greater exposure to the risk of accidents in occupational settings, particularly in rural activities and those involving the handling of sharp materials, increases susceptibility to infection. 

For women, the immunization strategy has proven more effective, particularly due to its focus on reducing neonatal tetanus. This approach, which prioritizes vaccination during pregnancy, has been widely adopted for several decades. As a result, the incidence rate of the disease among women has decreased over time, demonstrating there were advances in public vaccination policies ^12^^,^^18^. 

Evidence suggests a rising trend of tetanus among older adults, which is directly linked to lower vaccination rates and the age-related decline in immune function ^18^. Furthermore, age equal to or greater than 60 years was significantly associated with death, indicating greater vulnerability of this age group to fatal outcomes. 

Immunization is essential for preventing the disease, as well as for avoiding infection and progression to death, particularly among older adults. Indeed, older individuals often face greater exposure to risk factors such as comorbidities, medication use, and aging-related frailty, which reinforces the need for adequate vaccination strategies for this age group ^18^^,^^19^. Nevertheless, low adherence to immunization among adults, particularly older adults, combined with persistent gaps in public policies targeting this demographic, contributes to the higher frequency of preventable cases and the elevated case-fatality rate observed in this age group ^20^^,^^21^. In this context, the findings of this study underscore the importance of expanding vaccination strategies specifically targeted to adults and older adults.

Accidental tetanus primarily affects individuals engaged in rural labor. However, since the 1970s, Brazil has undergone a significant transformation and is no longer a predominantly rural country. This is evidenced in the present study, as most cases occurred in individuals residing in the metropolitan region of Fortaleza-a pattern similarly reported in other regions ^12^^,^^16^. 

The most frequent portal of entry was in the lower limbs, a finding that aligns with national ^22^ and international ^23^ data, reinforcing the need for preventive measures targeted at injuries in this area.

Clinical characteristics and their prevalence vary across different regions. For instance, in Pernambuco ^22^, between 2007 and 2015, the prevalence of clinical characteristics was similar, with trismus (n=117; 85.4%) being the most frequently recorded clinical manifestation, followed by muscle spasms (n=93; 67.9%) and nuchal rigidity (n=60; 43.8%). Also in Pernambuco, nuchal rigidity was a clinical manifestation associated with mortality due to its occurrence in more severe cases.

In this sample, tetanus case-fatality was 18.2%, lower than the national average of 27.3% ^3^, but higher than the rates reported in developed countries such as South Korea (10.2%, between 2011 and 2019) and the United States (13.2%, between 2001 and 2008) ^17^. The incidence and mortality of tetanus are linked to the region's socioeconomic development level, as countries with better sociodemographic indicators tend to exhibit lower mortality rates, as well as reduced age-specific incidence and years lived with disability rates. 

The quality of care, especially management in ICU settings, has a significant influence on case-fatality rates. Regions with low sociodemographic indices, such as Africa and Asia, often face higher mortality rates due to the unavailability of adequate equipment, invasive mechanical ventilation, and specialized multidisciplinary teams ^2^.

In Brazil, between 2007 and 2016, the Northeast region reported the highest number of tetanus deaths, coinciding with the region's lowest human development index, which encompasses several aspects, including life expectancy, education, income, and factors intrinsically related to the adoption of public policies ^10^. It is known that any wound is a potential entry point for Clostridium tetani. However, in Brazil, less than 50.0% of individuals with acute or chronic injuries seek care and prophylaxis for tetanus ^24^.

Considering that the individuals in this study are of working and economically active age, decreased muscle function and consequent functional impairment directly impact social and financial aspects of life, length of hospitalization, and rehabilitation costs, thereby overburdening the Brazilian National Health System ^20^.

The number of tetanus cases and tetanus-related deaths in Fortaleza between 2017 and 2024 shows fluctuations, with a decrease from 2017 to 2021, a peak in 2022 with nine cases, followed by a reduction in the two subsequent years. This increase observed in 2022, the year following the peak of the COVID-19 pandemic, may be related to the decline in vaccine coverage for immunopreventable diseases during the pandemic period ^25^. Moreover, it is possible that underreporting of tetanus cases occurred during the pandemic, particularly for milder clinical forms, due to the overburdening of health services and reduced surveillance capacity in several locations ^26^. 

Considering these findings, there is a clear need for future research to develop effective strategies for expanding vaccination coverage among adults and older adults, identify the primary barriers to immunization, and propose feasible solutions to promote greater adherence. It is recommended that health managers prioritize the creation of educational campaigns targeting this population, promote the training of professionals to actively address vaccination in Primary Health Care services, and ensure continuous and accessible provision of doses across different levels of care. It is also essential to strengthen systems for recording and monitoring vaccination coverage in order to identify risk groups and guide more effective interventions.

This study has limitations due to its retrospective design, small sample size, and reliance on data from medical records, which may introduce bias in data collection. Additionally, much of the evidence on accidental tetanus comes from older sources. Nevertheless, it was conducted in a referral hospital for infectious diseases in Ceará, which reinforces its clinical relevance. Future studies with larger samples are necessary to deepen the understanding of accidental tetanus management.

In conclusion, individuals with accidental tetanus admitted to the ICU were predominantly working-age men and agricultural workers, with incomplete elementary education and either no vaccination information or incomplete vaccination schedules. Being 60 years of age or older and smoking were significantly associated with a higher likelihood of death. These findings therefore reinforce the importance of identifying the risk profile and expanding vaccination coverage.

## Data Availability

Research data can be obtained upon request to the corresponding author.
