# Supplementary material for: Epidemiological profile of people with accidental tetanus admitted to the intensive care unit of a public hospital: a retrospective study, Fortaleza, 2017-2024
Source: Epidemiol Serv Saude. 2026 Apr 4;35:e20250232. doi: 10.1590/S2237-96222026v35e20250232.en (PMC13686401; doi:10.1590/S2237-96222026v35e20250232.en)
Supplement: Translation

# Translation: Perfil epidemiológico de pessoas com tétano acidental internadas em unidade de terapia intensiva em hospital público: estudo retrospectivo, Fortaleza, 2017-2024

**DOI:** 10.1590/S2237-96222026v35e20250232.pt

**Authors:** Lara Figueiredo Vasconcelos, Francisco Cleiton Ribeiro Freitas, João Pedro Nunes Leiros, Carlos Heitor Ribeiro dos Santos, Caio Douglas Guilherme Rodrigues, Riany De Sousa Sena

**Affiliations:** 1 Hospital São José de Doenças Infecciosas, Fortaleza, CE, Brasil Hospital São José de Doenças Infecciosas Fortaleza CE Brasil; 2 Hospital Geral Dr. Waldemar Alcântara, Fortaleza, CE, Brasil Hospital Geral Dr. Waldemar Alcântara Fortaleza CE Brasil; 3 Universidade de Fortaleza, Fortaleza, CE, Brasil Universidade de Fortaleza Fortaleza CE Brasil

**Keywords:** Tétano, Unidades de Terapia Intensiva, Perfil de Saúde, Hospitais Especializados, Estudos Transversais.

## Abstract

**Objetivo:**

Analisar o perfil epidemiológico de pessoas com tétano acidental internadas em unidade de terapia intensiva em Fortaleza no período 2017-2024.

**Métodos:**

Tratou-se de estudo de coorte retrospectivo, realizado em um hospital público do Ceará. Foram incluídos indivíduos com diagnóstico confirmado de tétano, idade ≥18 anos, independentemente do sexo, internados em unidade de terapia intensiva entre janeiro de 2017 e maio de 2024. Foi excluído um prontuário com dados sociodemográficos e clínicos insuficientes.

**Resultados:**

Foram incluídos no estudo 44 participantes, sendo a maioria do sexo masculino (n=41), com predomínio da faixa etária de 20-40 anos (n=20); casados (n=19), de raça/cor da pele parda (n=40) e com ensino fundamental incompleto (n=20). Houve predomínio daqueles que habitavam Fortaleza (n=18). Os trabalhadores da agropecuária (n=14) tiveram maior representatividade. As portas de entradas mais frequentes foram os pés (n=15). A maioria desconhecia o próprio esquema vacinal (n=26). A idade igual ou superior a 60 anos (*odds ratio*, OR 11,6; intervalos de confiança de 95%, IC95% 1,9; 70,2; p-valor 0,003) e o tabagismo (OR 9,8; IC95% 1,1; 88,2; p-valor 0,019) foram significativamente associados ao desfecho de óbito.

**Conclusão:**

Observou-se o predomínio de homens de idade produtiva, com ensino fundamental incompleto, e trabalhadores da agropecuária. A idade igual ou superior a 60 anos e o tabagismo estiveram significativamente associados a uma maior chance de óbito.

**Ethical aspects**

Esta pesquisa respeitou os princípios éticos, obtendo os seguintes dados de aprovação:

Comitê de ética em pesquisa Hospital São José de Doenças Infecciosas

Número do parecer: 6.847.296

Data de aprovação: 24/6/2024

Certificado de apresentação de apreciação ética: 78330624.2.0000.5044

Registro do consentimento livre e esclarecido: Dispensado.

## Introdução 

O tétano acidental é uma infecção grave, não transmissível, resultante da ação de exotoxinas liberadas pela bactéria *Clostridium tetani*^1^. Embora a incidência tenha diminuído globalmente, o tétano ainda representa considerável causa de morbimortalidade nos países em desenvolvimento e subdesenvolvidos, onde o acesso à vacinação e aos serviços de saúde é limitado ^2^. Entre 2014 e 2024, o Brasil registrou 2.385 casos confirmados de tétano acidental, com maior incidência nas regiões Nordeste (33,2%), Sudeste (21,4%) e Sul (21,3%). Em 2022, 2023 e 2024, a letalidade no Brasil foi 26,6%, 29,2% e 26,9%, valores superiores aos observados em países desenvolvidos como Japão (6,8%, entre 2010 e 2016) e Estados Unidos (13,2%, entre 2001 e 2008) ^3^^-^^5^.

A imunidade contra o tétano é adquirida por meio da vacinação. O Sistema Único de Saúde (SUS), segundo o Plano Nacional de Imunizações, oferece um esquema vacinal para o tétano iniciado com três doses no primeiro ano de vida, um reforço aos 15 meses e outro reforço aos 4 anos de idade. A partir daí, a vacinação é realizada a cada dez anos, ou a cada cinco anos em caso de ferimentos graves ^6^. Apesar de ser evitável pela vacinação, o tétano ainda evolui para formas graves, o que exige internação em unidade de terapia intensiva (UTI), sobretudo em populações mais vulneráveis ^2^^,^^7^.

A recomendação para o tratamento do tétano acidental é que as formas moderadas e graves sejam conduzidas em UTI para monitoramento contínuo e tratamento intensivo por equipe multidisciplinar capacitada, principalmente ao existir fator de risco de mau prognóstico como a disautonomia ^8^. No Brasil, no período 2007-2016, a duração média de internamento por tétano acidental foi de 17,1 dias, com o valor por internação em média de R$ 5.022,32 e o valor estimado de gastos por ano de R$ 9.852.868,52 ^9^. O tétano acidental apresenta elevada taxa de letalidade, o que contribui para o aumento do número de internações e, por consequência, dos custos elevados, sobrecarregando o sistema de saúde ^10^.

A persistência de casos graves revela notáveis déficits na Atenção Primária à Saúde, como falhas na vigilância ativa e na educação em saúde. Diversas barreiras estruturais e contextuais, como a distância de unidades de saúde, as dificuldades de transporte e a desarticulação entre os níveis de atenção, comprometem a efetividade das ações de prevenção e promoção da saúde. Torna-se imprescindível um planejamento em saúde que reconheça as especificidades territoriais e socioculturais dos usuários, permitindo a formulação de estratégias com foco na ampliação do acesso e na efetivação do cuidado integral ^11^.

A vulnerabilidade para o tétano não é homogeneamente distribuída e varia de acordo com fatores ocupacionais, ambientais e socioeconômicos ^2^. No Brasil, o tétano acidental tem mostrado uma tendência a afetar mais a população idosa e os trabalhadores rurais, devido à menor cobertura vacinal e à maior vulnerabilidade a acidentes. Isso é evidenciado pelo coeficiente de mortalidade que tem diminuído entre as faixas etárias, exceto entre as pessoas com mais de 60 anos ^12^. 

Com a diminuição da incidência da doença, podem ocorrer mudanças no perfil epidemiológico, o que demanda avaliação contínua para guiar políticas públicas e estratégias preventivas ^2^. Embora seja uma doença que pode ser eficazmente prevenida com vacinação e educação em saúde continuada, acessível e de baixo custo, o tétano acidental permanece um problema de saúde pública e traz repercussões sistêmicas, econômicas e sociais ^2^. Assim, a melhor compreensão sobre o perfil das pessoas acometidas pelo tétano pode ajudar a direcionar o planejamento e a execução de ações de políticas públicas de saúde e vigilância epidemiológica, além de estratégias preventivas e campanhas de vacinação. 

O objetivo deste estudo foi analisar o perfil epidemiológico de pessoas com tétano acidental internadas em UTI em um hospital público de Fortaleza entre 2017-2024.

## Métodos 

Delineamento

Tratou-se de estudo de coorte retrospectivo, com base em revisão de prontuários, realizado em um hospital público referência em doenças infecciosas no Ceará.

Contexto

O estudo foi realizado entre junho de 2023 e janeiro de 2025 em um hospital público cearense, referência em doenças infecciosas e integrante da rede SUS, que traz, em seu histórico, a participação no controle de graves doenças infecciosas e endêmicas no estado ^13^. Em 2025, a instituição conta com 120 leitos, sendo seis unidades de internamento e uma UTI. 

Participantes

A amostra incluiu pessoas com diagnóstico de tétano acidental confirmado e registrado nas fichas de notificações da unidade de vigilância epidemiológica. O diagnóstico foi estabelecido com base nas manifestações clínicas, como trismo, rigidez de nuca, crises de contraturas, riso sardônico, rigidez abdominal, opistótono e rigidez de membros. Outros critérios de inclusão foram: idade ≥18 anos, sexo (masculino, feminino) e necessidade de internação em UTI entre janeiro de 2017 e maio de 2024. Foi excluído um prontuário com dados sociodemográficos e clínicos insuficientes para coleta. 

Variáveis

As variáveis coletadas dos prontuários foram variáveis sociodemográficas e clínicas, como idade, sexo, estado civil, raça/cor da pele, escolaridade, residência, ocupação, índice de massa corporal e presença de comorbidade(s). Foram coletados dados referentes ao tétano, como porta de entrada, status do esquema vacinal, tempo de incubação, tempo de sintomas e desfecho clínico (mortalidade ou alta hospitalar).

Fonte de dados 

Os dados foram obtidos a partir das fichas de notificação da unidade de vigilância epidemiológica e dos prontuários e registros hospitalares. As informações foram coletadas por meio de formulário de dados, preenchido por um profissional da saúde, e tabeladas em uma planilha de Excel para, em seguida, serem analisadas. 

Viés 

O estudo possui risco de viés na coleta de informações por conta do delineamento retrospectivo, uma vez que os registros clínicos nem sempre são completos, padronizados ou precisos, podendo haver omissões ou erros de registro que comprometem a qualidade dos dados analisados. 

Tamanho do estudo

A amostra foi do tipo não aleatória, com base nos dados disponíveis nos prontuários eletrônicos durante a triagem inicial dos participantes.

Métodos estatísticos 

Os dados foram analisados utilizando o software Statistical Package for the Social Sciences (Chicago, Estados Unidos, versão 24). Inicialmente, foi realizada a análise descritiva, com apresentação de frequências absoluta e relativa para as variáveis categóricas, e média, desvio-padrão, mediana e intervalo interquartil para as variáveis numéricas. A normalidade da distribuição foi verificada por meio do teste de Shapiro-Wilk. Para avaliar os fatores associados ao desfecho (óbito), foram calculadas medidas de associação por meio da razão de chance (*odds ratio*, OR) com respectivos intervalos de confiança de 95% (IC95%). O valor de p-valor<0,05 foi considerado estatisticamente significante. 

## Resultados 

Foram notificadas 62 pessoas pela unidade de vigilância epidemiológica do hospital; desse total, 18 casos foram excluídos (Figura 1). A amostra final do estudo foi composta por 44 pessoas.

Observaram-se as características sociodemográficas dos participantes do estudo (Tabela 1). A maioria dos participantes era do sexo masculino (n=41) e das faixas etárias entre 40-59 anos (n=20) e 60+ (n=17), casados (n=19), da raça/cor da pele parda (n=40) e com ensino fundamental incompleto (n=20). Houve predomínio daqueles que habitavam Fortaleza (n=18) e o interior do Ceará (n=17). Os trabalhadores da área agropecuária (n=14) e desempregados (n=10) tiveram maior representatividade. A média do índice de massa corporal dos participantes foi 24,0 (20,7 kg/m²-26,0 kg/m²) kg/m². A maioria referiu ser etilista (n=27), enquanto a metade dos participantes era tabagista (n=22). A média de comorbidades foi 0,9±1,1, sendo as mais prevalentes hipertensão (n=13), diabetes (n=6) e doença obstrutiva crônica (n=4). 

**Figura 1:** Inclusão dos participantes no estudo. Fortaleza, 2017-2024 (n=62)

**Tabela 1:** Características sociodemográficas das pessoas com tétano acidental grave admitidas na unidade de terapia intensiva. Fortaleza, 2017-2024 (n=44)

Variáveis	n
Sexo masculino	41
Faixa etária (anos)	
20-39	7
40-59	20
≥60	17
Estado civil	
Casado	19
Solteiro	14
Não informado	6
Divorciado	3
Viúvo	2
Raça/cor da pele	
Parda	40
Preto	3
Branco	1
Escolaridade	
Ensino fundamental incompleto	20
Não sabe ler/escrever	11
Alfabetizado	3
Não informado	3
Ensino fundamental completo	3
Ensino médio incompleto	2
Ensino médio completo	2
Residência	
Fortaleza	18
Interior do Ceará	17
Região metropolitana de Fortaleza	8
Outro estado brasileiro	1
Ocupação	
Agropecuária	14
Desempregado	10
Trabalhador da construção civil	5
Vendedor	5
Autônomo	2
Aposentado	2
Coletor de resíduos	2
Serviços gerais	2
Confeiteira	1
Trabalhador(a) doméstico(a)	1
Etilismo	27
Tabagismo	22
Comorbidades	
Hipertensão	13
Diabetes	6
Doença pulmonar obstrutiva crônica	4
Dor lombar crônica	2
Enfisema pulmonar	2
Insuficiência renal crônica	2

Foram descritas as características clínicas relacionadas ao tétano acidental (Tabela 2). As portas de entrada mais frequentes foram: pés (n=15), pernas (n=10) e foco indefinido (n=7). A maior parte das pessoas desconhecia o próprio esquema vacinal (n=26), enquanto 11 relataram não terem sido vacinadas e sete apresentavam esquema vacinal incompleto. A mediana do tempo de incubação foi de dez (intervalo interquartil 7-15) dias, e o tempo de sintomas até a internação hospitalar, de três (intervalo interquartil 2-6) dias. Os sintomas registrados na admissão foram contraturas (n=41), trismo (n=40), disfagia (n=39), disartria (n=39), rigidez de membros (n=33), rigidez abdominal (n=29), rigidez de nuca (n=27), opistótono (n=13) e riso sardônico (n=6). Em relação ao desfecho hospitalar, 34 pessoas receberam alta hospitalar, oito evoluíram para óbito e duas foram transferidas para outra instituição.

Observou-se a evolução do número de casos de tétano e óbitos por tétano em Fortaleza no período 2017-2024 (Figura 2). Ocorreu um pico de casos de tétano em 2022, com nove registros, seguido de redução nos dois anos subsequentes. Em relação aos óbitos, o ano de 2022 também apresentou o maior número: quatro mortes. Ao longo do período analisado, notou-se variação tanto no número absoluto de casos quanto na letalidade, com destaque para 2020 e 2024. Nesses anos, embora os casos tenham sido relativamente baixos, a proporção de óbitos em relação aos casos foi elevada.

A idade igual ou superior a 60 anos (OR 11,6; IC95% 1,9; 70,2; p-valor 0,003) e o tabagismo (OR 9,8; IC95% 1,1; 88,2; p-valor 0,019) foram significativamente associados ao desfecho de óbito (Tabela 3).

**Tabela 2:** Características relacionadas às pessoas com tétano acidental admitidas na unidade de terapia intensiva. Fortaleza, 2017-2024 (n=44)

Variáveis	n
Porta de entrada	
Pés	15
Pernas	10
Indefinido	7
Dentário	4
Mãos	3
Braços	3
Cabeça/pescoço	1
Coluna	1
Esquema vacinal	
Não informado/desconhecido	26
Não vacinado	11
Plano incompleto	7
Sintomas na admissão	
Contraturas	41
Trismo	40
Disfagia	39
Disartria	39
Rigidez de membros	33
Rigidez abdominal	29
Rigidez de nuca	27
Opistótono	13
Riso sardônico	6
Desfecho clínico na internação hospitalar	
Alta hospitalar	34
Óbito	8
Transferência para outra instituição	2

**Figura 2:** Número de casos de tétano e óbitos por tétano. Fortaleza, 2017-2024

**Tabela 3:** Razões de chances (*odds ratio*, OR) e intervalos de confiança de 95% (IC95%) para óbito por tétano acidental, segundo variáveis do estudo. Fortaleza, 2017-2024 (n=42)

Variáveis	Óbito	Alta hospitalar	OR	(IC95%)	p-valor
Idade (anos)					
≥60	6	7	11,6	(1,9; 70,2)	0,003
<60	2	27			
Sexo			2,3	(0,2; 28,9)	0,513
Feminino	1	2			
Masculino	7	32			
Tabagismo					
Sim	4	5	9,8	(1,1; 88,2)	0,019
Não	4	30			
Tempo de sintomas (dias)					
≥7	4	16	3,4	(0,3; 42,4)	0,337
<7	2	10			
Uso de bloqueador neuromuscular					
Sim	8	23	1,3	(1,09; 1,6)	0,063
Não	0	11			
Disautonomia			1,8	(0,2; 17,3)	0,600
Sim	3	5			
Não	5	29			

## Discussão 

Neste estudo, observou-se que o tétano acidental grave acometeu predominantemente homens e indivíduos de 40-59 anos ou 60 anos ou mais. Esses indivíduos eram, em sua maioria, moradores de Fortaleza, casados, de raça/cor da pele parda, com ensino fundamental incompleto e trabalhadores da área agropecuária. A maioria desconhecia o próprio esquema vacinal ou não era vacinado contra o tétano, além de se declarar etilista e tabagista. As portas de entrada mais frequentes para o tétano foram pés, pernas e foco indefinido. Os sintomas foram contraturas, trismo, disfagia e disartria. A idade igual ou superior a 60 anos e o tabagismo estiveram significativamente associados a uma maior chance de óbito.

O perfil das pessoas deste estudo foi semelhante ao descrito em outras cidades do Nordeste, como em Alagoas, entre 2007 e 2017 ^14^, onde a maioria dos casos foi em pessoas do sexo masculino (91,0%), na faixa etária de 18-49 anos (67,2%), trabalhadores da agropecuária (27,8%) e com residência em região urbana (83,1%). No período 2006-2018, na Bahia ^15^, a prevalência dos casos foi em homens (83,3%), na faixa etária de 35-49 anos (30,3%), pardos (38,6%), com ensino fundamental incompleto (30,3%) e residência na capital (33,3%). A ocupação mais predominante nessa localidade foi a de lavradores (n=36, 27,3%) e pedreiros (n=26; 19,7%), porém neste estudo a maioria dos registros da amostra não tinha dados sobre a ocupação (37,9%) introduzindo viés ao resultado. Apesar da heterogeneidade socioeconômica do Brasil, o perfil das pessoas acometidas pelo tétano acidental entre 1990 e 2009 em São Paulo ^12^ e entre 2008 e 2018 em Santa Catarina ^16^ também foi similar a este estudo. 

Esse perfil é diferente daqueles de outros países como Coreia do Sul, França e Itália, já que a maior incidência do tétano acidental é no sexo feminino. Isso se dá, possivelmente, devido à maior cobertura vacinal em homens realizada durante o serviço militar compulsório ^17^.

A prevalência mais elevada da doença entre o sexo masculino, no Brasil, pode ser atribuída a diversos fatores, sendo o principal deles a baixa adesão às campanhas de vacinação. Esse contexto resulta em uma cobertura vacinal inferior, o que, por sua vez, contribui para a maior vulnerabilidade a doenças evitáveis, como o tétano. Além disso, a maior exposição aos riscos de acidentes em ambientes ocupacionais, especialmente em atividades rurais e que envolvem o manuseio de materiais cortantes, aumenta a suscetibilidade à infecção. 

No sexo feminino, a estratégia de imunização se mostra mais eficaz, especialmente com o foco na redução do tétano neonatal. Essa abordagem, que prioriza a vacinação durante o período gestacional, tem sido amplamente adotada há várias décadas. Como resultado, a taxa de incidência da doença nas mulheres tem diminuído ao longo do tempo, refletindo os avanços nas políticas públicas voltadas à vacinação ^12^^,^^18^. 

Observou-se a crescente senilização do tétano acidental diretamente relacionada à diminuição da cobertura vacinal e à redução da resposta imunológica que acompanha o envelhecimento ^18^. Observou-se também que a idade igual ou superior a 60 anos foi significativamente associada ao óbito, indicando maior vulnerabilidade dessa faixa etária ao desfecho fatal. 

A imunização é fator essencial para a prevenção da doença, o estabelecimento da infecção e a evolução para óbito, especialmente entre indivíduos com idade mais avançada. De fato, os idosos frequentemente enfrentam maior exposição a fatores de risco, como a presença de comorbidades, o uso de medicamentos e a fragilidade decorrente do envelhecimento, o que reforça a necessidade de estratégias de vacinação adequadas para esse grupo etário ^18^^,^^19^. No entanto, a baixa adesão à imunização entre adultos, particularmente os idosos, associada às lacunas persistentes nas políticas públicas direcionadas a essa população, contribui para a maior frequência de casos evitáveis e para a elevada letalidade observada nessa população ^20^^,^^21^. Nesse contexto, os achados deste estudo reforçam a importância da ampliação de estratégias específicas de vacinação para adultos e idosos.

O tétano acidental tem como fator de risco a população em atividade laboral rural, porém, a partir da década de 1970, o Brasil iniciou um processo de transformação e deixou de ser predominantemente rural. Isso é evidenciado neste estudo, visto que a maioria dos casos foi em indivíduos que residiam na região metropolitana de Fortaleza - e é apresentado de forma similar em outras regiões ^12^^,^^16^. 

A porta de entrada mais frequente foi em membros inferiores, achado semelhante no Brasil ^22^ e em outros países ^23^, o que reforça a necessidade de medidas preventivas voltadas para ferimentos nessa região. 

As características clínicas e sua prevalência variam em diferentes regiões. Em Pernambuco ^22^, no período 2007-2015, a prevalência das características clínicas foi similar, em que o trismo (n=117; 85,4%) foi a manifestação clínica mais registrada, seguida da contratura muscular (n=93; 67,9%) e rigidez de nuca (n=60; 43,8%). Nessa mesma localidade, a rigidez de nuca foi uma manifestação clínica que apresentou associação com a mortalidade devido à sua ocorrência em casos mais graves.

Nesta amostra, a letalidade por tétano foi 18,2%, inferior à média nacional de 27,3% ^3^, mas superior às taxas relatadas em países desenvolvidos, como Coreia do Sul (10,2%, entre 2011 e 2019) e Estados Unidos (13,2%, entre 2001 e 2008) ^17^. A incidência e a mortalidade do tétano são associadas ao nível de desenvolvimento socioeconômico da região, pois países com melhores índices sociodemográficos tendem a apresentar menores taxas de mortalidade, bem como taxas mais baixas de incidência por idade e anos vividos com incapacidade. 

A qualidade da assistência, especialmente no manejo em ambiente de UTI, exerce influência significativa sobre a letalidade. Regiões com baixos índices sociodemográficos, como África e Ásia, frequentemente enfrentam maiores taxas de mortalidade, devido à indisponibilidade de equipamentos, ventilação mecânica invasiva e equipe multiprofissional especializada ^2^.

No Brasil, entre 2007 e 2016, a região Nordeste apresentou o maior número de óbitos por tétano associado ao menor índice de desenvolvimento humano regional, que aborda diversos aspectos como expectativa de vida, educação, renda e fatores intrinsecamente ligados à adesão de políticas públicas ^10^. Sabe-se que qualquer tipo de ferida é um local de entrada em potencial para o *Clostridium tetani*, mas no Brasil menos de 50,0% das pessoas com lesões agudas ou crônicas procuram atendimento e profilaxia para o tétano ^24^.

Considerando que os indivíduos deste estudo estão em idade produtiva para o trabalho e economicamente ativa, a diminuição da função muscular e consequente funcionalidade impacta diretamente os aspectos sociais e financeiros do indivíduo, o tempo de internamento e os custos para reabilitação sobrecarregando o SUS ^20^.

O número de casos de tétano e dos óbitos associados à doença em Fortaleza entre 2017 e 2024 revela flutuações, observando-se queda entre 2017 e 2021, pico de casos em 2022, com nove registros, seguido por redução nos dois anos subsequentes. Esse aumento observado em 2022, ano posterior ao pico da pandemia de covid-19, pode estar relacionado à queda das coberturas vacinais de doenças imunopreveníveis durante o período pandêmico ^25^. Ademais, é possível que, durante a pandemia, tenha ocorrido subnotificações de casos de tétano, especialmente das formas clínicas menos graves, em decorrência da sobrecarga dos serviços de saúde e da redução da capacidade de vigilância em diversas localidades ^26^. 

Considerando estes achados, observa-se a necessidade de futuras pesquisas voltadas ao desenvolvimento de estratégias eficazes para ampliar a cobertura vacinal entre adultos e idosos, identificando as principais barreiras à imunização e propondo soluções viáveis para promover a maior adesão. Recomenda-se que gestores de saúde priorizem a criação de campanhas educativas voltadas para esse público, promovam a capacitação de profissionais para a abordagem ativa da vacinação em serviços de Atenção Primária e garantam a oferta contínua e acessível das doses nos diversos níveis de atenção. Também é fundamental o fortalecimento de sistemas de registro e monitoramento da cobertura vacinal, a fim de identificar grupos de risco e orientar intervenções mais eficazes.

Este estudo possui limitações por ser retrospectivo, com amostra pequena e com base em dados de prontuários médicos, o que pode introduzir viés na coleta de informações, além de muitas evidências sobre o tétano acidental serem de fontes mais antigas. No entanto, foi conduzido em um hospital de referência para doenças infecciosas no Ceará, o que reforça sua relevância clínica. Estudos futuros com amostras maiores são necessários para aprofundar o conhecimento sobre o manejo do tétano acidental.

Por fim, conclui-se que as pessoas com tétano acidental internadas em UTI eram predominantemente homens de idade produtiva e trabalhadores da agropecuária, com ensino fundamental incompleto e sem informação sobre vacinação ou com esquema vacinal incompleto. A idade igual ou superior a 60 anos e o tabagismo estiveram significativamente associados a uma maior chance de óbito. Assim, estes achados reforçam a importância da identificação do perfil de risco e da ampliação da cobertura vacinal.

## Data Availability

Os dados da pesquisa podem ser obtidos mediante solicitação ao autor correspondente.
